# *Williamsia* spp. are emerging opportunistic bacteria

**DOI:** 10.1016/j.nmni.2017.11.002

**Published:** 2017-11-24

**Authors:** Masoud Keikha

**Affiliations:** Department of Microbiology, School of Medicine, Isfahan University of Medical Sciences, Isfahan, Iran

**Keywords:** 16S rRNA, isolation, molecular identification, phenotypic test, Williamsia

Actinomycetes that have mycolic acid in the cell walls have been classified under genera such as *Corynebacterium*, *Gordonia*, *Mycobacterium*, *Nocardia*, *Rhodococcus*, *Tsukamurella*, *Skermania* and *Williamsia* [1]. *Williamsia* was introduced in 1999 by Kämpfer et al. [2] to accommodate actinomycetes with atypical cell morphology and mycolic acids with 50 to 56 carbon chain lengths between the genera of *Rhodococcus* and *Gordonia.* Members of the genus *Williamsia* are Gram-positive, aerobic, rod and coccoid shaped, smooth and orange-red pigmented (in some species) colonies and not acid-fast bacteria, and consist of straight-chain saturated, unsaturated and 10-methyl-branched components that currently comprise 11 recognized species which have been isolated from human clinical specimens and environmental resources [3], [4].

According to reports, *Williamsia deligens*, *W. muralis* and *W. serinedens* have been isolated from immunocompromised patients with diabetes mellitus, as well as elderly patients. The most commonly reported sources of *Williamsia* infection include pulmonary infection [5], bacteraemia [4], endophthalmitis [3] and perinatal sepsis [6]. In addition, evidence indicates that *Williamsia serinedens* is able to grow in oil-contaminated soil; this bacterium is likely to be effective in the biodegradation process and in the decomposition of industrial pollution from soil [7]. Members of aerobic actinomycetes are increasing by discovering new species, and clinical microbiologists will face problems identifying them. Phenotypic tests are unable to identify actinomycetes and differentiate them from each other. Conventional tests include urea hydrolysis, adenine, casein, elastin, aesculin, gelatin, guanine, hypoxanthine, testosterone, tyrosine and xanthine. l-Alanine, acetamide, arginine, gelatin, ornithine, proline and serine are utilized as carbon and nitrogen sources. Adsorption of acetate, 2,3-butandiol, citrate, mannitol, paraffin, sorbitol, trehalose, adonitol, adipate, isoamyl alcohol, l-arabinose, cellobiose, meso-erythritol, m-hydroxybenzoate, p-hydroxybenzoate, myo-inositol, lactate, melezitose, 1,2-propandiol and carbohydrates as carbon sources are usually labor intensive and time consuming. Further, many reports of *Williamsia* infection are usually misdiagnosed as *Rhodococcus* and other actinomycete infections; thus, analyzing whole fatty-acid cell walls via molecular methods (such as DNA hybridization techniques and 16S rRNA gene sequencing) is a rapid, accurate and reliable method for identifying actinomycete infections, especially *Williamsia* infection [2], [4], [5], [8].

Not much information about susceptibility to antimicrobial drugs is available for the genus *Williamsia.* The best antibiotic antimicrobial test is microbroth dilution, as presented by the Clinical and Laboratory Standards Institute [9]. *Williamsia* spp. are resistant to ampicillin, oxacillin, erythromycin and trimethoprim/sulfamethoxazole but susceptible to amoxicillin/clavulanate, cefotaxime, imipenem, ciprofloxacin, tobramycin, gentamicin, cotrimoxazole, amikacin, linezolid, meropenem, penicillin G and vancomycin [5], [6].

In summary, our knowledge of the isolation and identification of different *Williamsia* spp. is limited. On the basis of previous reports, this group of aerobic actinomycetes comprise opportunistic microorganisms that cause a variety infections in immunocompromised patients. Given that the pathogenic virulence factors and antibiotic resistance of *Williamsia* are unknown, it is necessary to clarify its features to permit appropriate identification, treatment and management.

## Conflict of interest

None declared.
